# Direct Measure of the Local Concentration of Pyrenyl Groups in Pyrene-Labeled Dendrons Derived from the Rate of Fluorescence Collisional Quenching

**DOI:** 10.3390/polym12122919

**Published:** 2020-12-05

**Authors:** Janine L. Thoma, Stuart A. McNelles, Alex Adronov, Jean Duhamel

**Affiliations:** 1 Department of Chemistry, Institute for Polymer Research, Waterloo Institute for Nanotechnology, University of Waterloo, Waterloo, ON N2L 3G1, Canada; janine.lydia.thoma@uwaterloo.ca; 2 Department of Chemistry and Chemical Biology, Brockhouse Institute for Materials Research, McMaster University, 1280 Main St. W., Hamilton, ON L8S 4M1, Canada; mcnellsa@mcmaster.ca (S.A.M.); adronov@mcmaster.ca (A.A.)

**Keywords:** pyrene excimer fluorescence, pyrene-labeled dendrimers, model-free analysis

## Abstract

The model-free analysis (MFA) was applied to measure the average rate constant (<*k*>) for pyrene excimer formation (PEF) in a series of pyrene-labeled dendrons referred to as Py_x_-G(*N*), where *x* (= 2^N^) is the number of pyrenyl labels born by a dendron of generation *N* ranging from 1 to 6. <*k*> was measured in four different solvents, namely tetrahydrofuran (THF), toluene, *N*,*N*-dimethylformamide (DMF), and dimethylsulfoxide (DMSO). <*k*> was found to increase linearly with increasing local pyrene concentration ([*Py*]_loc_), where [*Py*]_loc_ had been determined mathematically for the Py_x_-G(*N*) dendrons. The slope of each straight line changed with the nature of the solvent and represented *k*_diff_, the bimolecular rate constant for PEF. *k*_diff_ depended on the solvent viscosity (*η*) and the probability (*p*) for PEF upon encounter between an excited and a ground-state pyrene. In a same solvent, *k*_diff_ for the Py_x_-G(*N*) dendrons was about 360 ± 30 times smaller than *k*_diff_ obtained for ethyl 4-(1-pyrene)butyrate (PyBE), a pyrene model compound similar to the pyrene derivative used to label the dendrons. The massive decrease in *k*_diff_ observed for the Py_x_-G(*N*) samples reflected the massive loss in mobility experienced by the pyrenyl labels after being covalently attached onto a macromolecule compared to freely diffusing PyBE. Interestingly, the *k*_diff_ values obtained for the Py_x_-G(*N*) dendrons and the PyBE model compound followed similar trends as a function of solvent, indicating that the difference in behavior between the *k*_diff_ values obtained in different solvents were merely due to the changes in the *η* and *p* values between the solvents. Normalizing the <*k*> values obtained with the Py_x_-G(*N*) dendrons by the *k*_diff_ values obtained for PyBE in the same solvents accounted for changes in *η* and *p,* resulting in a master curve upon plotting <*k*>/(*f*_diff_ × *k*_diff_) as a function of [*Py*]_loc_, where *f*_diff_ was introduced to account for some pyrene aggregation in the higher generation dendron (Py_64_-G(6)). This result demonstrates that <*k*> represents a direct measure of [*Py*]_loc_ in pyrene-labeled macromolecules.

## 1. Introduction

Techniques such as viscometry, light scattering (LS), and small angle X-ray (SAXS) or neutron (SANS) scattering have traditionally played a critical role in the characterization of macromolecules due to their ability to determine the internal density of macromolecules in solution. Since the internal density of a macromolecule can be related to its volume after the molecular weight has been determined, such measurements provide a means to assess the dimensions of macromolecules in solution. For instance, the existence of excluded volume experienced by macromolecules in solution is readily detected by a marked decrease in the internal density of macromolecules, which can be mathematically predicted and experimentally confirmed through intrinsic viscosity ([*η*]) and LS measurements [1]. These measurements are typically conducted with polymer concentrations in the 1–10 g/L range, a concentration range that is suitable for well-soluble samples, but that can lead to aggregation and challenging data analysis for less soluble macromolecules.

In contrast, fluorescence is better known for its ability to probe fast photochemical processes, which has led to the implementation of many fluorescence-based applications to characterize the internal dynamics of macromolecules in solution [2]. In a typical fluorescence collisional quenching (FCQ) experiment, where quenching occurs solely upon contact between the excited dye and its quencher, a macromolecule is covalently labeled with a dye and its quencher at two specific positions, followed by the acquisition of the monoexponential fluorescence decay of the fluorescently labeled macromolecule. Analysis of the decay yields the decay time (*τ*) corresponding to the quenching of the dye by the quencher, which is equal to (1/ *τ*_o_ + *k*_diff_ × [*Q*]_loc_)^−1^, where *τ*_o_, *k*_diff_, and [*Q*]_loc_ are the dye natural lifetime, the bimolecular quenching rate constant describing the diffusive encounters between the excited dye and the quencher, and the quencher concentration experienced locally by the excited dye, respectively. Because [*Q*]_loc_ is deemed impossible to measure experimentally, the fluorescence decay analysis yields the product *k*_diff_ × [*Q*]_loc_, which is referred to as the pseudo-unimolecular rate constant *k*. Information about the internal dynamics of the macromolecule is retrieved from *k*, a larger *k* reflecting a more flexible macromolecule. Yet, this interpretation of *k* overlooks the fact that *k* is not only a function of *k*_diff_ but also of [*Q*]_loc_, which is related to the internal density of the macromolecule, since the quencher is covalently attached to the macromolecule.

The difficulty in establishing the relationship between *k* and [*Q*]_loc_ for fluorescently labeled macromolecules is rooted in a number of technical and theoretical hurdles that must be overcome, as described in an earlier review [3]. First, most photophysical processes like FCQ occur over short distances of less than 5 nm [3]. This constraint requires that the dye and quencher be relatively close to each other, limiting the application of FCQ experiments to oligomers, not macromolecules. To study macromolecules by FCQ and resolve this first hurdle, the dye and quencher must be brought closer to each other, typically by increasing the number of dyes and quenchers attached to the macromolecule. Unfortunately, this practice leads to the second hurdle of FCQ experiments, whereby each polymer segment spanning a dye and a quencher results in a different quenching rate constant, which leads to a complex distribution of quenching rate constant (*k*_i_) [3]. In turn, the fluorescence decay of the randomly labeled macromolecules turns into a sum of exponentials associated with a distribution of decay times *τ*_i_ equal to (1/*τ*_o_ + *k*_i_)^−1^. Since no multiexponential decay analysis can resolve all the *τ*_i_ values resulting from the distribution of the *k*_i_ rate constants, the average rate constant for quenching <*k*> is typically determined. The relationship between <*k*> and [*Q*]_loc_ now needs to be established, and this represents the third hurdle in FCQ experiments because [*Q*]_loc_ is difficult to predict for a macromolecule labeled with more than one dye and one quencher [3].

For reasons that have been presented in several reviews [3,4,5], excimer formation upon encounter between an excited and a ground-state pyrenyl group covalently attached onto a macromolecule is a well-known and often used FCQ application to probe the internal dynamics of macromolecules. The multiexponential fluorescence decays acquired with macromolecules labeled with more than two pyrenes can be satisfyingly fit according to the model-free analysis (MFA), which yields the average rate constant <*k*> of pyrene excimer formation (PEF) [3,6]. In this case, the ground-state pyrenes act as quenchers, and <*k*> is related to the local pyrene concentration ([*Py*]_loc_) experienced by an excited pyrene according to Equation (1). Equation (1) is well accepted in the literature because it has been shown to hold for homogeneous pyrene solutions [7], it agrees with the results obtained with end-labeled monodisperse polymers [8,9], and it has been predicted for polymers randomly labeled with pyrenes [10]. The MFA has been shown to be a superior analytical tool [11] compared to other procedures that have been applied previously to study the fluorescence of pyrene-labeled dendrimers [12,13,14,15,16,17,18,19,20,21,22,23,24,25,26,27,28], as was reported in an earlier review [4]. Yet, validation of Equation (1) is essential to demonstrate that FCQ experiments such as those based on PEF yield parameters that report on [*Py*]_loc_, and thus on the internal density of the macromolecule onto which the pyrenyl labels are attached.

(1) $$\langle k\rangle =k_{diff}\times {\left[Py\right]}_{loc}$$

The only study to have indicated that Equation (1) is valid was conducted in tetrahydrofuran (THF) with a series of pyrene labeled dendrons referred to as Py*_x_*-G(*N*). The Py_x_-G(*N*) dendrons were prepared with a 2,2-bis(hydroxymethyl) propionic acid backbone, the generation number (*N*) of the dendrons ranged from 1 to 6, and *x* (= 2^N^) represented the number of pyrenyl labels covalently attached to the terminal ends of a dendron of generation *N* [29]. The published derivation of the average squared end-to-end distance, <*L*_Py_^2^>^1/2^, of the highly branched Py_x_-G(*N*) dendrons was employed to estimate [*Py*]_loc_ for each Py_x_-G(*N*) dendron. The MFA of the fluorescence decays acquired with the Py_x_-G(*N*) dendrons yielded <*k*>, which was found to increase linearly with increasing [*Py*]_loc_ as predicted by Equation (1). This original study is now extended to the three additional solvents toluene, *N*,*N*-dimethylformamide (DMF), and dimethylsulfoxide (DMSO), which provide, after including THF, a series of four solvents with a broad range of polarity and viscosity. In turn, polarity and viscosity affected the probability (*p*) of forming an excimer and the diffusion coefficient (*D*) of the pyrene labels in different manner, which affected the results obtained by PEF. Fortunately, these effects could be accounted for by studying PEF with homogeneous solutions of ethyl 4-(1-pyrene)butyrate (PyBE) in the same solvents, which provided a means to assess how *k*_diff_ in Equation (1) was affected by solvent polarity and viscosity. After accounting for the changes in *k*_diff_ due to solvent polarity and viscosity, all <*k*> vs. [*Py*]_loc_ plots obtained for the Py_x_-G(*N*) samples in the different solvents merged into a single master curve, thus demonstrating the validity and generality of Equation (1).

## 2. Materials and Methods

### 2.1. Materials

The six 2,2-bis(hydroxymethyl)propionic acid backbone dendrimers bearing pyrenyl labels at each terminal end have been previously synthesized, and their synthesis and characterization in THF can be reviewed in a previous publication [29]. The chemical structure of the pyrene-labeled dendrons (Py_x_-G(*N*)) used in this study is provided in Table 1.

Dichloromethane (DCM, ≥ 99.8%), *N*,*N*’-dicyclohexylcarbodiimide (DCC, ≥ 99.0%), 4-(dimethylamino)pyridine (DMAP, ≥ 99%), *N*,*N*-dimethylformamide (DMF, ≥ 99.8%), dimethylsulfoxide (DMSO, ≥ 99.9%), ethanol (reagent grade), 1-pyrenebutyric acid (97%), sodium hydroxide (≥97.0%), and sodium sulfate (anhydrous ≥ 99.0%) were obtained from Sigma (Markham, ON, Canada). Sodium bicarbonate (≥99.7%), tetrahydrofuran optima (≥99.9%), and toluene (distilled in glass) were supplied by VWR (Mississauga, ON, Canada), Fisher Scientific (Ottawa, ON, Canada), and Caledon Laboratories (Halton Hills, ON, Canada), respectively. All chemicals were used as received.

### 2.2. Synthesis of Ethyl 4-(1-pyrene)butyrate (PyBE)

**Scheme 1:**
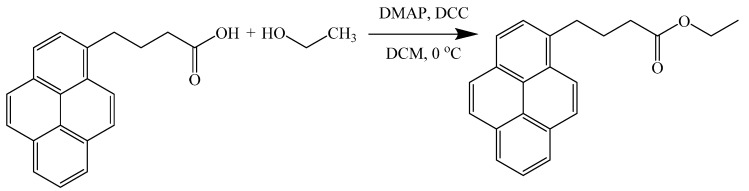
Chemical reaction scheme of the synthesis of the model compound ethyl 4-(1-pyrene)butyrate (PyBE).

The synthesis of the model compound ethyl 4-(1-pyrene)butyrate (PyBE) follows the reaction scheme outlined in Scheme 1 and is described in more detail hereafter. Freshly distilled DCM (25 mL) was added to a 50 mL round bottom flask (RBF) equipped with a magnetic stir bar. 1-Pyrenebutyric acid (1.00 g, 3.5 mmol), ethanol (1.60 g, 34.7 mmol), and DMAP (0.08 g, 0.7 mmol) were added to the RBF. The RBF was then placed in an ice water bath under a gentle flow of nitrogen (Praxair, 4.0). DCC (0.72 g, 3.5 mmol) was dissolved in 5 mL of DCM, and the solution was added dropwise to the RBF over 30 min. The reaction was left stirring under a nitrogen atmosphere at room temperature for 18 h. The next day, the reaction solution was filtered using suction filtration to remove the urea precipitate. The reaction solution was then washed with 0.5 M HCl, a saturated solution of sodium bicarbonate, and a saturated solution of sodium chloride. The organic layer was extracted after each wash and dried with sodium sulfate. Silica gel chromatography was then used to purify PyBE using a 1:10 ethyl acetate:hexane mixture. The final product was isolated as a colourless solid and dried in vacuo overnight (0.68 g, 62%). The ^1^H NMR spectrum is shown in Figure S1 in the Supplementary Materials (SM), and its molar absorbance coefficient at 344 nm equals 42,600 ± 140 M^−1^·cm^−1^ in THF.

### 2.3. UV–Vis Spectroscopy

All absorption measurements were carried out on a Varian Cary 100 Bio spectrophotometer (Varian, Palo Alto, CA, USA). The Py(*x*)-G(*N*) solutions were prepared with a pyrene concentration of 2.5 × 10^−6^ M equivalent to an optical density of 0.1. The absorbance measurements were made using a quartz cuvette with a 10 mm path length.

### 2.4. Steady-State Fluorometer

All steady-state fluorescence (SSF) measurements were performed on a QM-400 spectrofluorometer equipped with a Xenon arc lamp (HORIBA, London, ON, Canada). The different pyrene solutions in organic solvents were degassed for 30–45 min depending on the organic solvent. and their spectra were acquired with a 344 nm excitation wavelength and over wavelengths ranging from 350 to 600 nm. The SSF measurements with the Py_x_-G(*N*) dendrons were conducted using the conventional right-angle geometry, and the measurements carried out with more concentrated solutions of the PyBE model compound were performed using the front face geometry to avoid the inner filter effect and reabsorption. The intensity of the excimer emission, *I*_E_, was divided by the intensity of the monomer emission, *I*_M_, to obtain the *I*_E_/*I*_M_ ratio, which is a measure of PEF efficiency. *I*_M_ was calculated by integrating the area under the first fluorescence peak of the monomer fluorescence spectrum from *λ*_0-0_ – 4 nm to *λ*_0-0_ + 4 nm, where *λ*_0-0_ corresponds to the wavelength of the 0-0 transition of the pyrene derivative in a given solvent. Since *I*_E_ was much less sensitive to solvent differences, it was calculated by integrating the excimer fluorescence intensity under the spectrum from 500 to 530 nm.

### 2.5. Time-Resolved Fluorometer

A FluoroHub fluorometer (HORIBA, Piscataway, NJ, USA) equipped with a DeltaDiode at 336 nm was used to acquire the monomer and excimer time-resolved fluorescence (TRF) decays of the degassed solutions of the Py_x_-G(*N*) dendrons and PyBE in the different solvents at 375 and 510 nm with a 370 and 490 nm cut off filter, respectively. The cut off filters prevented stray light from reaching the fluorescence detector. All decays had 20,000 counts at their maximum and were acquired over 1024 channels with times-per-channel of 0.435, 0.0514 or 0.102 ns/ch. A 2.04 ns/ch time-per-channel was used for the fluorescence decays of 2.5 × 10^−6^ M dilute solutions of the PyBE and Py_1_-G(0) model compounds acquired to determine the natural lifetime (*τ*_M_) of the pyrene derivatives. The instrument response function (IRF) was obtained with an aluminum reflective monolith by setting the emission wavelength at 336 nm. The IRF was then convoluted with the mathematical expressions for the time-dependent concentration of the pyrene monomer and excimer given in SI as Equations (S1) and (S2) for the MFA [3,6] and as Equations (S3) and (S4) for the Birks scheme analysis [7,9]. The parameters in Equations (S1)–(S4) were optimized according to the Marquardt–Levenberg algorithm [30]. The quality of the fits was gauged from a low *χ* ^2^ (< 1.20) and residuals and autocorrelation of the residuals randomly distributed around zero.

### 2.6. Model-Free Analysis (MFA) of the Fluorescence Decays

The model-free analysis (MFA) is a global analysis tool which can be used to simultaneously fit a monomer and excimer decay [3,4,5,6,29]. The MFA can be used to fit the decays of any molecule or macromolecule labeled with the chromophore pyrene. When pyrene becomes excited by a photon of light, it can decay to the ground state using one of two pathways. It can either fluoresce with its monomeric lifetime, *τ*_M_, or it can encounter another ground-state pyrene and form one of two excimers. An excimer can be the result of the proper (E0) and improper (D) stacking of two pyrenyl groups and they have their own lifetime, *τ*_E0_ and *τ*_D_, respectively. Three different pyrene species are expected to co-exist in solution, namely those pyrenes that are isolated in solution and cannot form excimer (*Py*_free_*), form an excimer through diffusive encounters (*Py*_diff_*), and are pre-aggregated (E0* or D*). The MFA yields the molar fractions *f*_free_, *f*_diff_, and *f*_agg_ (= *f*_E0_ + *f*_D_) of the pyrene species *Py*_free_*, *Py*_diff_*, E0*, and D*, respectively. In some instances, a contribution from residual scattering or possibly short-lived pyrene dimers is seen in the excimer decays, and an exponential with a short lifetime (*τ*_S_) of 4 ns is added to the expression of the excimer decay during the decay analysis. More information about the details of the MFA can be found as SM, along with the equations used to fit the pyrene monomer and excimer fluorescence decays.

Using the decay times, *τ*_i_, and pre-exponential factors, *a*_i_, calculated by the MFA, the average lifetime, <*τ*>, as well as the average rate constant of excimer formation, <*k*>, can be calculated as shown using Equations (2) and (3), respectively.

(2) $$<\tau >\;=\;\sum_{i=1}^{3}a_{i}\tau_{i}$$

(3) $$<k>\;=\;\frac{1}{<\tau >}\;-\;\frac{1}{\tau_{M}}$$

Using the molar fractions of the different pyrene species along with <*τ*> and <*k*>, the *I*_E_/*I*_M_^TRF^ ratio representing the absolute *I*_E_/*I*_M_ ratio shown in Equation (4) can be calculated [31].

(4) $${I_{E}/I_{M}}^{TRF}\;=\;\frac{(f_{diffE0}\tau_{E0}\;+\;f_{diffD}\tau_{D})<\tau ><k>\;+\;f_{E0}\tau_{E0}\;+\;f_{D}\tau_{D}}{f_{diff}<\tau >\;+\;f_{free}\tau_{M}}$$

### 2.7. Birks Scheme Analysis of the Fluorescence Decays

The pyrene monomer and excimer fluorescence decays of PyBE were also fitted globally with Equations (S3) and (S4), respectively. In this case, the excimer is assumed to be produced by diffusive encounters between an excited and a ground-state pyrene label with a single rate constant *k*_diff_. The excimer can then fluoresce with its natural lifetime *τ*_E0_ or dissociate by returning an excited and ground-state pyrene with a dissociation rate constant *k*_-1_. Global analysis of the decays yields *k*_diff_ × [*PyBE*], *k*_-1_, and *τ*_E0_, where [*PyBE*] is the concentration of the PyBE model compound [7,9,32].

## 3. Results and Discussion

The average squared end-to-end distance, <*L*_Py_^2^>^1/2^, of a series of 2,2-bis(hydroxymethyl)propionic acid backbone dendrons labeled with 1-pyrenebutyric acid at their terminal ends (Py_x_-G(*N*)) is shown in Equation (5), which has been derived in an earlier publication [29]. The parameters *a* (= 6) and *b* (= 8) in Equation (5) represent the number of atoms connecting the pyrene label to the first junction point and the number of atoms resulting from the incorporation of each bis(hydroxymethyl)propionic acid in the construct, respectively. In Equation (5), *l* represents the projection of a C–C bond length taken to equal 0.125 nm.

(5) $$<L_{Py}^{2}>\;=\;l^{2}\left(1+2a+b\frac{N\times 2^{N}-2^{N+1}+2}{2^{N}-1}\right)$$

[*Py*]_loc_ could then be determined with Equation (6), where *N*_A_ is the Avogadro number and the term (2^N^–1) represents the number of ground-state pyrenes in the Py_x_-G(*N*) dendron after considering that one pyrenyl label must be excited in a PEF experiment. The [*Py*]_loc_ concentrations corresponding to the different Py_x_-G(*N*) dendrons are provided in Table 1. The very large [*Py*]_loc_ concentrations reported in Table 1 are a result of the short distances separating the ends of the dendrons in comparison to their overall size. For instance, a linear chain made of 16 bonds would have an end-to-end distance equal to 16^0.5^ × *l* = 0.5 nm using an *l* value of 0.125 nm. This end-to-end distance would result in a molar concentration for one chain end according to Equation (6) equal to 44 M, which is much larger than many organic solvents, but is a consequence of dealing with the end-to-end distance of a molecule instead of its overall size that is much larger. With their much higher number of chain ends, the Py_x_-G(*N*) dendrons have considerably higher local concentrations of chain ends, as reported in Table 1.

(6) $${\left[Py\right]}_{loc}\;=\;\frac{4}{3}\pi \frac{2^{N}\;-\;1}{{\left(<L_{Py}^{2}{>}^{1/2}/2\right)}^{3}\times N_{A}}$$

**Figure 1:**
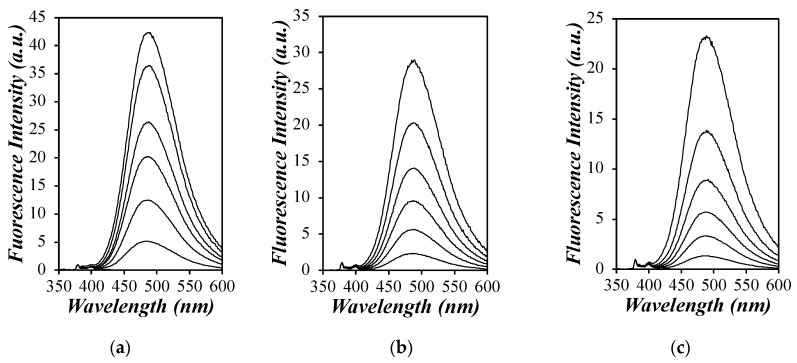
Steady-state fluorescence (SSF) spectra of the Py*_x_*-G(*N*) dendrons normalized at the 0-0 transition of pyrene (~378 nm) in (**a**) toluene, (**b**) *N*,*N*-dimethylformamide (DMF), and (**c**) dimethylsulfoxide (DMSO). From top to bottom: Py_64_-G(6), Py_32_-G(5), Py_16_-G(4), Py_8_-G(3), Py_4_-G(2), and Py_2_-G(1). *λ*_ex_ = 344 nm, [Py] = 2.5 × 10^−6^ M.

The steady-state fluorescence (SSF) spectra for each Py*_x_*-G(*N*) sample were acquired in the different solvents and are shown in Figure 1 for toluene, DMF, and DMSO. Py_2_-G(1), which contains two pyrenyl groups, produces the least amount of excimer in each solvent, and PEF was found to increase with generation number and the number of pyrenyl groups present in a given Py_x_-G(*N*) dendron, thus reflecting a direct relationship between the PEF efficiency and [*Py*]_loc_. The SSF spectra shown in Figure 1 are typical of a pyrene-labeled macromolecule, and the PEF efficiency can be better quantified by considering the *I*_E_/*I*_M_ ratio, which is proportional to *k*_diff_ and [*Py*]_loc_, as shown in Equation (7). The expression of *k*_diff_ is given in Equation (8), where *R*, *D*, and *p* are the molecular diameter of pyrene, twice the diffusion coefficient of pyrene, and the probability of having PEF upon a diffusive encounter between an excited and a ground-state pyrene, respectively [2]. The expression of the diffusion coefficient (*D*_Py_) for one pyrene molecule is given in Equation (9), where *k*_B_, *T*, *η*, and *R*_h_ are the Boltzmann constant, the absolute temperature, the solvent viscosity, and the hydrodynamic radius of the dye, respectively. Based on Equations (7)–(9), *k*_diff_, and thus the *I*_E_/*I*_M_ ratio, is proportional to the ratio *p*/*η*, which depends critically on the dielectric constant (reported at 20 °C) and viscosity (reported at 25 °C) of the solvent, which equal 2.4 and 0.56 mPa.s, 7.5 and 0.46 mPa.s, 38 and 0.79 mPa.s, and 47 and 1.99 mPa.s for toluene, THF, DMF, and DMSO, respectively [33].

(7) $$\frac{I_{E}}{I_{M}}\propto k_{\mathrm{diff}}\times {\left[Py\right]}_{\mathrm{loc}}$$

(8) $$k_{\mathrm{diff}}=4\pi N_{A}RDp$$

(9) $$D=\frac{k_{B}T}{6\pi \eta R_{h}}$$

**Figure 2:**
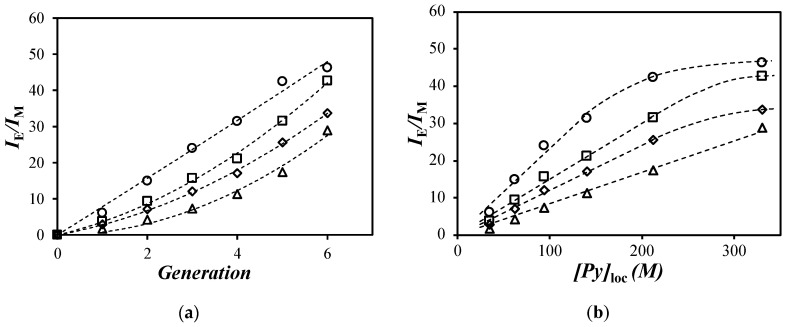
Plot of the *I*_E_/*I*_M_ ratios for each Py*_x_*-G(*N*) dendron in (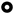) toluene, (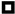) tetrahydrofuran (THF) [29], (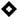) DMF, and (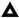) DMSO versus (**a**) generation number and (**b**) the [*Py*]_loc_.

The *I*_E_/*I*_M_ ratios of the Py*_x_*-G(*N*) dendrons were plotted in Figure 2a for each solvent. The *I*_E_/*I*_M_ ratio increased with increasing generation number. However, and as predicted by Equations (7)–(9), the *I*_E_/*I*_M_ ratio did not depend solely on the viscosity of the solvent. For instance, toluene with a viscosity higher than THF was found to form excimer more efficiently, followed by THF, DMF, and finally DMSO. THF, which is more than four times less viscous than DMSO, yielded *I*_E_/*I*_M_ ratios that were only 2.4-fold larger than those obtained in DMSO. This behaviour was a result of the different *p* values taken in Equation (8) for different solvents. The *I*_E_/*I*_M_ ratios were also plotted as a function of [*Py*]_loc_ in Figure 2b. Fairly linear trends were obtained between *I*_E_/*I*_M_ and [*Py*]_loc_ up to Py_32_-G(5), but clear deviations from linearity were observed for Py_64_-G(6). The linear increase in *I*_E_/*I*_M_ with increasing [*Py*]_loc_ agreed with the behavior expected from Equation (7).

The somewhat odd behavior observed in Figure 2b for Py_64_-G(*N*), whereby the *I*_E_/*I*_M_ ratio is smaller than anticipated, has two most plausible causes. One would be the presence of a small but strongly emissive amount of unattached pyrenyl labels, which would artificially increase the pyrene monomer emission, thus reducing the *I*_E_/*I*_M_ ratio as was illustrated in an earlier publication [11]. The second would be the enhanced crowding of the terminal pyrenyl groups for the Py_64_-G(6) sample leading to a reduction in the number of pyrenyl groups available for diffusive PEF, and thus resulting in less efficient PEF as was suggested earlier for the trends obtained in THF [29]. To address these concerns as well as the somewhat erratic behavior shown in Figure 2b where each solvent yielded a different trend, a more detailed analysis of the fluorescence data is required by applying the model-free analysis (MFA) to the fluorescence decays of the Py_x_-G(*N*) samples. The MFA dissects the PEF process into its dynamic and structural components given by the average rate constant <*k*> for PEF (see Equation (1)) and the molar fractions of the different pyrene species contributing to PEF, respectively.

**Figure 3:**
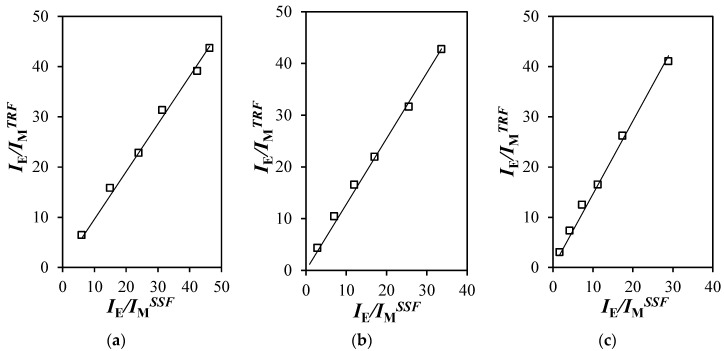
Plot of *I*_E_/*I*_M_^TRF^ versus *I*_E_/*I*_M_^SSF^ for the Py*_x_*-G(*N*) samples in (**a**) toluene, (**b**) DMF, and (**c**) DMSO.

The TRF decays of each Py*_x_*-G(*N*) dendron were acquired in toluene, DMF, and DMSO and then analysed using the model-free analysis (MFA). The global MFA of the decays gave excellent fits with a *χ*^2^ ≤ 1.2, and residuals and autocorrelation residuals randomly distributed around zero. A sample fit is shown in Figure S2. A detailed explanation of the MFA can be found as Supplementary Materials (SM) as well as all the parameters retrieved from the fits in Tables S3–S5. The MFA parameters were used to calculate <*k*> from Equation (3) and the molar fractions of the different pyrene species. These molar fractions *f*_free_, *f*_diff_, and *f*_agg_ represent the pyrenes that do not form excimer in solution and are usually free unattached pyrene derivatives, form excimer through diffusive encounters, and form excimer through direct excitation of a pyrene aggregate, respectively. To ensure that the parameters retrieved from the MFA provided an accurate representation of the fluorescence spectra shown in Figure 2, the parameters retrieved from the MFA of the decays were used to calculate the absolute *I*_E_/*I*_M_^TRF^ ratios using Equation (4). The absolute *I*_E_/*I*_M_^TRF^ ratios calculated from Equation (4) should be proportional to the relative *I*_E_/*I*_M_^SSF^ ratios obtained from the analysis of the SSF spectra. The linear relationship observed in Figure 3 between the *I*_E_/*I*_M_^TRF^ and *I*_E_/*I*_M_^SSF^ ratios demonstrated that the MFA parameters retrieved from the global analysis of the fluorescence decays agreed with the analysis of the fluoresce intensities in the fluorescence spectra. The average rate constant <*k*> could now be studied in more detail.

**Figure 4:**
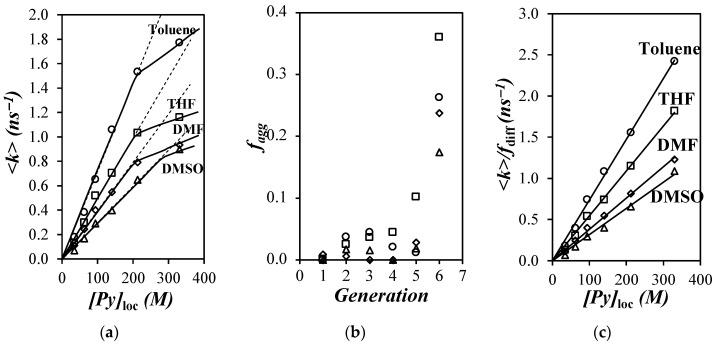
Plot of (**a**) <*k*> versus [*Py*]_loc_ for the Py*_x_*-G(*N*) dendrons, (**b**) *f*_agg_ calculated by the model-free analysis (MFA) for each generation, and (**c**) <*k*>/*f*_diff_ versus [*Py*]_loc_ for the Py*_x_*-G(*N*) dendrons in (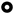) toluene, (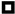) THF [29], (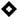) DMF, and (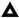) DMSO.

**Figure 5:**
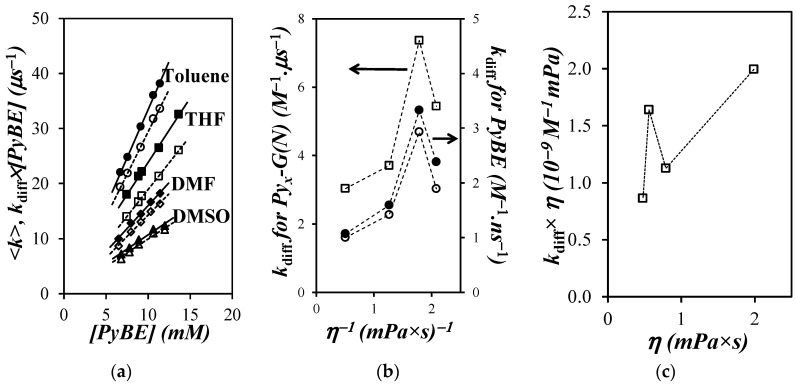
(**a**) <*k*> and *k*_diff_ × [*PyBE*] versus [PyBE] in (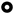,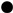) toluene, (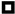,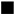) THF, (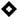,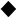) DMF, and (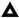,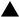) DMSO; solid and hollow symbols are for the fluorescence decay analysis according to the Birks scheme and MFA, respectively. (**b**) Plot of *k*_diff_ for (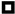) the Py*_x_*-G(*N*) dendrons and the PyBE model compound when the fluorescence decays were fitted according to (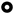) the MFA and (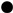) the Birks scheme. (**c**) *k*_diff_ × *η* of PyBE versus solvent viscosity.

Figure 4a shows a plot of <*k*> vs. [*Py*]_loc_ for the Py_x_-G(*N*) samples in the three solvents. As for the *I*_E_/*I*_M_^SSF^ ratios in Figure 2b, <*k*> increased linearly with increasing [*Py*]_loc_ for *N* values ranging from 1 to 5 but showed a clear downward curvature for Py_64_-G(6). The same behavior had been observed earlier in THF, and this behavior had been attributed to increased aggregation of the pyrenyl labels, which lowered the local concentration of active pyrenyl labels that could form excimer by diffusive encounters between an excited and a ground-state pyrene [29]. The notion that the pyrenyl labels were strongly aggregated in the Py_64_-G(6) sample was easily illustrated by plotting the molar fraction of aggregated pyrenes (*f*_agg_) as a function of the generation number in Figure 4b. While the Py_x_-G(*N*) samples with 1 ≤ *N* ≤ 5 yielded *f*_agg_ values that were smaller than 0.06, *f*_agg_ for the Py_64_-G(6) sample took a value that was larger than 0.17 in all solvents studied. Since <*k*> is unaffected by the presence of free pyrene derivatives, this result confirmed our earlier analysis [29] that the unexpected behavior observed for Py_64_-G(6) in Figure 2b and Figure 5a was probably a consequence of extensive aggregation of the pyrenyl labels due to overcrowding. Dividing <*k*> by the molar fraction of pyrenyl groups, which form excimer exclusively though diffusive encounters (*f*_diff_) [29], accounts for this artefact by increasing <*k*> to the value that it should take if all its pyrenyl groups were dissolved in the solution, as assumed by Equation (6) for the calculation of [*Py*]_loc_.

This correction was applied, and the ratio <*k*>/*f*_diff_ was plotted as a function of [*Py*]_loc_ in Figure 4c. The linear relationships obtained in the plots shown in Figure 4c for all Py*_x_*-G(*N*) dendrons in toluene, THF, DMF, and DMSO validated the proposal that the unexpected behavior observed for the *I*_E_/*I*_M_^SSF^ and <*k*> parameters in Figure 2b and Figure 5a was due to extensive aggregation of the pyrenyl groups in Py_64_-G(6). Similarly to the trends obtained in Figure 2b with the *I*_E_/*I*_M_^SSF^ ratios, <*k*> was found to be larger in the more viscous toluene than in THF. In an effort to identify the origin of this phenomenon and assess whether it was general or specific to the family of Py_x_-G(*N*) dendrons, the PEF process was investigated further with ethyl 4-(1-pyrene)butyrate (PyBE), which was used as a model compound, since it resembles the pyrene derivative covalently bound to the Py_x_-G(*N*) samples.

The fluorescence spectra of PyBE solutions with concentrations ranging from 6 to 14 mM were acquired in toluene, THF, DMF, and DMSO and are presented in Figure S3 in SM. The fluorescence spectra showed the expected spectral features of a pyrene derivative forming excimer, with a set of sharp peaks observed between 370 and 410 nm indicative of the pyrene monomer and a broad structureless emission centered at 480 nm for the excimer. Increasing the PyBE concentration resulted in increased pyrene–pyrene encounters that resulted in increased PEF. The fluorescence spectra shown in Figure S3 were analyzed to obtain their *I*_E_/*I*_M_^SSF^ ratios, which were plotted as a function of the PyBE concentration in Figure S4. The plots were linear as expected for PEF, but they also displayed the same unexpected trend observed with the Py_x_-G(*N*) dendrons that showed more viscous toluene yielding larger *I*_E_/*I*_M_^SSF^ ratios than less viscous THF.

The fluorescence decays of the pyrene monomer and excimer of all the PyBE solutions were acquired and analyzed according to the Birks scheme and the MFA. The parameters retrieved from the Birks scheme analysis and MFA for the PyBE solutions are listed in Tables S1 and S2, respectively. The fluorescence decay fits were excellent with the MFA in all solvents and for the Birks scheme in toluene and THF, but showed some slight deviations at the early times in DMF and DMSO, possibly due to the increased viscosity of the two latter solvents. Nevertheless, the product *k*_diff_ × [*PyBE*] obtained from the Birks scheme analysis of the fluorescence decays and <*k*> obtained from the MFA are plotted as a function of the PyBE concentration in Figure 5a. Both <*k*> and *k*_diff_ × [*PyBE*] increased linearly with increasing PyBE concentration, with <*k*> being always slightly smaller than the product *k*_diff_ × [*PyBE*], probably a consequence of the different assumptions made for the derivation of each model. The Birks scheme assumes that the pyrene excimer dissociates with a dissociation rate constant that is typically small compared to 1/*τ*_E0_, as found experimentally in Table S1, whereas the MFA neglects the excimer dissociation altogether. The Birks scheme also assumes a single rate constant for excimer formation, whereas the MFA assumes that the excimer can be formed by several rate constants. The poorer fits obtained for the fit of the fluorescence decays with the Birks scheme analysis in DMF and DMSO suggest that the MFA is a more robust analysis method. Nevertheless, the slopes of the plots of <*k*> and *k*_diff_ × [*PyBE*] as a function of PyBE concentration in Figure 5a and of <*k*> versus [*Py*]_loc_ in Figure 4c yielded the bimolecular rate constant (*k*_diff_) for diffusive encounters between two pyrene molecules for PyBE and the pyrenyl labels of the Py_x_-G(*N*) dendrons, respectively. They are plotted in Figure 5b as a function of the inverse of the solution viscosity.

The three plots for *k*_diff_ show some striking similarities with a sharp maximum for a 1/*η* value of 1.79 (mPa·s)^−1^ obtained in toluene. The common behavior observed for the pyrenyl labels of the Py_x_-G(*N*) dendrons and PyBE demonstrates that this behavior is a result of the pyrene dye and not due to the fact that it is attached onto the dendrons. *k*_diff_ was found to be 360 ± 30 times larger for the PyBE model compound than for the pyrenyl labels covalently attached to the dendrons. This massive reduction in *k*_diff_ reflects the equally massive loss in mobility experienced by a pyrene derivative upon covalent attachment to a macromolecule versus the same pyrene derivative freely diffusing in solution. Another important feature of Figure 5b is that *k*_diff_ is not inversely proportional to the solution viscosity, because PEF depends also on the probability (*p*) of forming an excimer upon encounter between two pyrene moieties, as discussed in Equations (7)–(9). This aspect is further illustrated with Figure 5c, which shows the product *η* × *k*_diff_, which is proportional to *p*, plotted as a function of the solvent viscosity. For the four solvents investigated, *η* × *k*_diff_ fluctuates from 0.86 × 10^9^ M^−1^·mPa in THF to 2.00 × 10^9^ M^−1^·mPa in DMSO. Additionally, the *η* × *k*_diff_ value of 1.13 × 10^9^ M^−1^·mPa for DMF was almost half that of 2.00 × 10^9^ M^−1^·mPa in DMSO, suggesting a similar relationship for *p* as had been suggested earlier with two other pyrene model compounds, namely 1-pyrenemethylacetamide [34] and *n*-butyl-1-pyreneacetamide [35].

**Figure 6:**
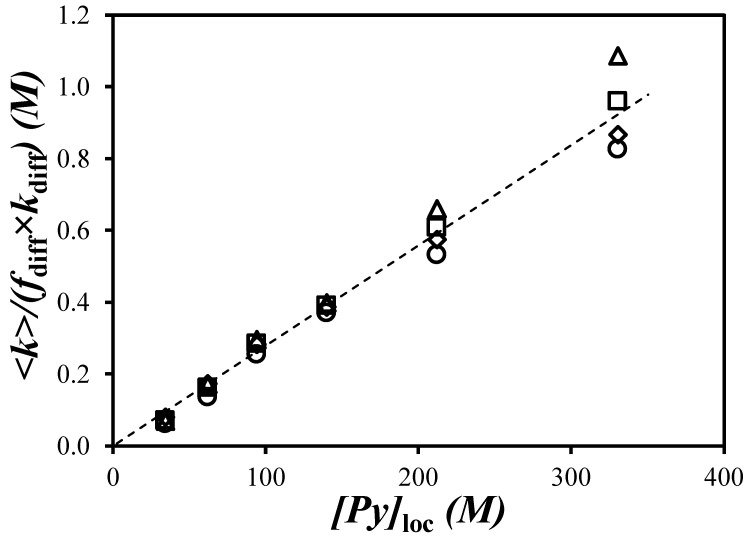
Plot of <*k*>/(*f*_diff_ × *k*_diff_) as a function of [*Py*]_loc_ for the Py_x_-G(*N*) dendrons where the <*k*> values obtained for the dendrimers in a given solvent are divided by *f*_diff_ to account for possible aggregation of the pyrenyl labels and *k*_diff_ obtained from the MFA of the PyBE fluorescence decays in the same solvent to account for the *p*/*η* term in Equations (8)–(9); (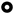) toluene, (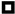) THF [29], (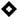) DMF, and (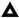) DMSO.

For the sake of consistency, the <*k*>/*f*_diff_ values obtained for the Py_x_-G(*N*) dendrons in different solvents were divided by the *k*_diff_ values of 1.90, 2.93, 1.42, and 1.00 M^−1^·ns^−1^ obtained from the MFA of the PyBE decays acquired in THF, toluene, DMF, and DMSO (see Figure 5b) to account for the changes in the ratio *p*/ *η* between the different solvents. The quantity <*k*>/(*f*_diff_ × *k*_diff_) was plotted as a function of [*Py*]_loc_ in Figure 6 for the four solvents considered. All data points in Figure 4c collapsed along a master line in Figure 6, indicating that this correction accounted for the different *p/η* values of the solvents. The data points showed more scatter for the higher generation dendrimers, possibly because the pyrenyl labels were more aggregated, but also because the larger dendrons might have been subject to more excluded volume effects, which are not accounted for by the derivation of Equation (4). The smaller dendrons are likelier to be devoid of solvent effects, and their behavior is well-described by Equation (4), which assumes that each chain segment behaves like a Gaussian chain. Finally, the straight line obtained between <*k*>/(*f*_diff_ × *k*_diff_) and [*Py*]_loc_ demonstrates that <*k*> responds directly to [*Py*]_loc_ for the pyrene-labeled dendrons regardless of the nature of the solvent.

## 4. Conclusions

A series of six pyrene end-labeled dendrons (Py*_x_*-G(*N*)) were studied in toluene, THF, DMF, and DMSO. These four solvents had different polarity and viscosity, which affected PEF through diffusive encounters between the pyrenyl labels differently. Accounting for the ratio *p*/*η* by dividing the <*k*> values obtained for each Py_x_-G(*N*) samples in different solvents by *k*_diff_ obtained for the model compound PyBE in the same solvents yielded a straight line between <*k*>/(*f*_diff_ × *k*_diff_) and [*Py*]_loc_. This relationship was found to hold regardless of the nature of the solvents, particularly for the smaller dendrons that are devoid of solvent effects. It also demonstrates that <*k*> obtained from the MFA of the fluorescence decays acquired with pyrene-labeled macromolecules depends directly on [*Py*]_loc_ and can thus be used to probe the internal density of macromolecules in the same manner as other techniques like intrinsic viscosity or static light scattering do. The main difference between PEF and these other techniques is that it takes advantage of the extreme sensitivity of fluorescence, which allows fluorescence measurements to be performed with PyLM solutions as low as 1 mg/L, two to three orders of magnitude lower than all other standard techniques that are used to characterize macromolecules in solution.

## Figures and Tables

**Table 1:** Chemical structure and [*Py*]_loc_ for each Py*_x_*-G(*N*) dendron [29].

**Sample**	**Py_2_-G(1)**	**Py_4_-G(2)**	**Py_8_-G(3)**	**Py_16_-G(4)**
Structure	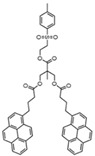	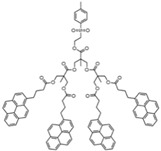	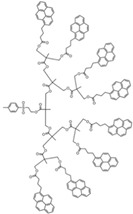	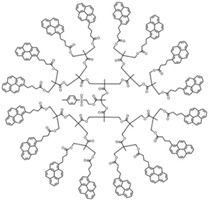
[*Py*]_loc_ (M)	34	62	94	140
**Sample**	**Py_32_-G(5)**		**Py_64_-G(6)**	
Structure	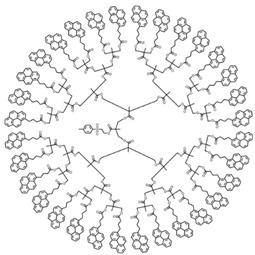		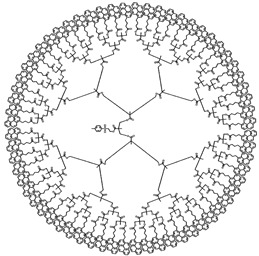	
[*Py*]_loc_ (M)	212		330	

## Supplementary Materials

The following are available online at https://www.mdpi.com/2073-4360/12/12/2919/s1: **Scheme S1:** Pyrene excimer formation according to the Birks scheme; **Figure S1:** ^1^H-NMR spectrum of ethyl 4-(1-pyrene)butyrate (PyBE). (300 MHz, CDCl3): δ 1.30 (t, 3H), 2.23 (p, 2H), 2.48 (t, 2H), 3.42 (t, 2H), 4.18 (q, 2H), 7.82–8.41 (m, 9H); **Figure S2:** MFA of the (A) monomer (λ_em_ = 375 nm) and (B) excimer (λ_em_ = 510 nm) decay of Py_32_-G(5) in degassed DMSO. χ^2^ = 1.13, λ_ex_ = 344 nm; **Figure S3:** SSF spectrum of ethyl 4-pyrenylbutanoate in (A) THF, (B) toluene, (C) DMF, and (D) DMSO; **Figure S4:** Plot of the *I*_E_/*I*_M_ ratio versus concentration of PyBE in () toluene, () THF, () DMF, and () DMSO; **Table S1:** Parameters retrieved from the global Birks scheme analysis of both the monomer and excimer decays of ethyl 4-(1-pyrene)butyrate in degassed toluene (Tol), *N*,*N*-dimethylformamide (DMF), dimethylsulfoxide (DMSO), and tetrahydrofuran (THF). Analysis program: globirks32bg; **Table S2:** Parameters retrieved from the MFA (analysis program: sumegs10bg) of both the monomer and excimer decays of ethyl 4-(1-pyrene)butyrate in degassed tetrahydrofuran (THF), degassed toluene (Tol), degassed dimethylformamide (DMF), and degassed dimethylsulfoxide (DMSO); **Table S3:** Parameters retrieved from the MFA (analysis programs: sumegs14bg or sumegs33bg-4) of the monomer decays of the Py_x_-G(*N*) dendrons in degassed toluene (Tol), degassed dimethylformamide (DMF), and degassed dimethylsulfoxide (DMSO); **Table S4:** Parameters retrieved from the MFA of the excimer decays of the Py_x_-G(*N*) dendrons in degassed toluene (Tol), degassed dimethylformamide (DMF), and degassed dimethylsulfoxide (DMSO); **Table S5:** Molar fractions obtained from the MFA of the Py_x_-G(*N*) dendrons in degassed toluene (Tol), degassed dimethylformamide (DMF), and degassed dimethylsulfoxide (DMSO).
