# Supplementary material for: Direct Measure of the Local Concentration of Pyrenyl Groups in Pyrene-Labeled Dendrons Derived from the Rate of Fluorescence Collisional Quenching
Source: Polymers (Basel). 2020 Dec 5;12(12):2919. doi: 10.3390/polym12122919 (PMC7762123; doi:10.3390/polym12122919)
Supplement: Supplementary file 1 [file polymers-12-02919-s001.pdf]

polymers

Supporting Information

Direct Measure of the Local Concentration of Pyrenyl Groups in Pyrene-Labeled Dendrons Derived from the Rate of Fluorescence Collisional Quenching

Janine L. Thoma [1], Stuart A. McNelles [2], Alex Adronov [2], and Jean Duhamel [1,*]

[1] Department of Chemistry, Institute for Polymer Research, Waterloo Institute for Nanotechnology, University of Waterloo, Waterloo, ON N2L 3G1, Canada; janine.lydia.thoma@uwaterloo.ca

[2] Department of Chemistry and Chemical Biology, Brockhouse Institute for Materials Research, McMaster University, 1280 Main St. W., Hamilton, Ontario, Canada L8S 4M1; mcnellsa@mcmaster.ca (S.A.M.); adronov@mcmaster.ca (A.A.)

* Correspondence: jduhamel@uwaterloo.ca

Global Model-Free Analysis (MFA) of the Monomer and Excimer Fluorescence Decays

Equations S1 and S2 were used to fit the monomer and excimer fluorescence decays globally according to the model-free analysis (MFA). As explained in the main text, pyrene excimer formation (PEF) is assumed to occur with a distribution of rate constants that results in a distribution of decay times (τ_i) that is handled by a sum of exponentials with decay times τ_i and pre-exponential factors (a_i). Three species are expected to be present in a solution of a pyrene derivative leading to PEF. These are the pyrenes that are isolated, cannot form excimer, and behave as if they free in solution (Py_{free}*), form excimer by diffusive encounters (Py_{diff}*), and are aggregated as $E0$* and EL*, where $E0$* and EL* correspond to pyrene aggregates that are well- and poorly stacked and emit with their lifetime τ_{E0} and τ_{EL}, respectively. The pyrene species that generate an excimer $E0$* or EL* by diffusion are referred to as Py_{diffE0}* and Py_{diffEL}*, respectively.

$$[Py^*]_{(t)} = ([Py^*_{diffE0}]_{(t=0)} + [Py^*_{diffD}]_{(t=0)}) \times \sum_{i=1}^{n} a_i \times \exp(-t/\tau_i) + [Py^*_{free}]_{(t=0)} \times \exp(-t/\tau_M) \quad \text{(S1)}$$

$$[E^*]_{(t)} = -[Py^*_{diffE0}]_{(t=0)} \times \sum_{i=1}^{n} a_i \frac{\frac{1}{\tau_i} - \frac{1}{\tau_M}}{\frac{1}{\tau_i} - \frac{1}{\tau_{E0}}} \exp(-t/\tau_i)$$

$$+ \left([E0^*]_{(t=0)} + [Py^*_{diffE0}]_{(t=0)} \times \sum_{i=1}^{n} a_i \frac{\frac{1}{\tau_i} - \frac{1}{\tau_M}}{\frac{1}{\tau_i} - \frac{1}{\tau_{E0}}} \right) \times \exp(-t/\tau_{E0})$$

$$-[Py^*_{diffEL}]_{(t=0)} \times \sum_{i=1}^{n} a_i \frac{\frac{1}{\tau_i} - \frac{1}{\tau_M}}{\frac{1}{\tau_i} - \frac{1}{\tau_{EL}}} \exp(-t/\tau_i)$$

$$+\left([EL^*]_{(t=0)} + [Py^*_{diffEL}]_{(t=0)} \times \sum_{i=1}^{n} a_i \frac{\frac{1}{\tau_i} - \frac{1}{\tau_M}}{\frac{1}{\tau_i} - \frac{1}{\tau_{EL}}} \right) \times \exp(-t/\tau_{EL}) + [ES^*]_{(t=0)} \times \exp(-t/\tau_S)$$

(S2)

The MFA program retrieves parameters that yields the molar fractions f_{diffE0}, f_{diffEL}, f_{free}, f_{E0}, and f_{EL} of the species Py_{diffE0}^*, Py_{diffEL}^*, Py_{free}^*, $E0^*$, and EL^*, respectively. The state of the different pyrene molecules, which are forming by diffusive encounters, isolated, and aggregated, is then represented by the molar fractions f_{diff} (= $f_{\text{diffE0}} + f_{\text{diffEL}}$), f_{free}, and f_{agg} (= $f_{\text{E0}} + f_{\text{EL}}$), respectively.

Global Birks Scheme Analysis of the Monomer and Excimer Fluorescence Decays

The kinetic scheme representing pyrene excimer formation according to the Birks scheme is described in Scheme S1.

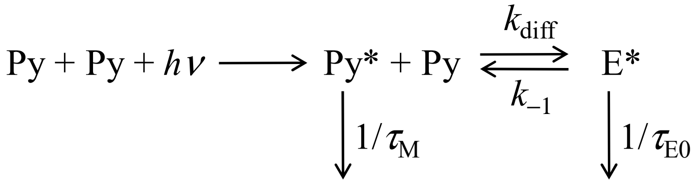

Scheme S1. Pyrene excimer formation according to the Birks scheme.

Absorption of a photon by a ground-state pyrene in Scheme S1 results in an excited pyrene that can either fluoresce with its natural lifetime τ_M or diffusively encounter a ground-state pyrene to form an excimer ($E0^*$) with a rate constant k_{diff}. The excimer can either fluoresce with its lifetime τ_{E0} or dissociate with a rate constant k_{-1}. According to Scheme S1, the equations for the time-dependent concentrations of the pyrene monomer and excimer are presented in Equations S3–S6, where $X = k_{\text{diff}} \times [Py] + \tau_M^{-1}$ and $Y = k_{-1} + \tau_{\text{E0}}^{-1}$.

$$[Py^*] = \frac{[Py^*]_o}{\sqrt{(X-Y)^2 + 4k_{diff}k_{-1}}} \left[\left(X - \tau_2^{-1} \right) \times \exp(-t/\tau_1) - (X - \tau_1^{-1}) \times \exp(-t/\tau_2) \right] \quad \text{(S3)}$$

$$[E^*] = \frac{k_1[Py^*]_o}{\sqrt{(X-Y)^2 + 4k_{diff}k_{-1}}} \left[-\exp(-t/\tau_1) + \exp(-t/\tau_2) \right] \quad \text{(S4)}$$

$$\tau_1^{-1} = \frac{X + Y + \sqrt{(X-Y)^2 + 4k_{diff}k_{-1}}}{2} \quad \text{(S5)}$$

$$\tau_2^{-1} = \frac{X + Y - \sqrt{(X-Y)^2 + 4k_{diff}k_{-1}}}{2} \quad \text{(S6)}$$

^{1}H-NMR Spectrum

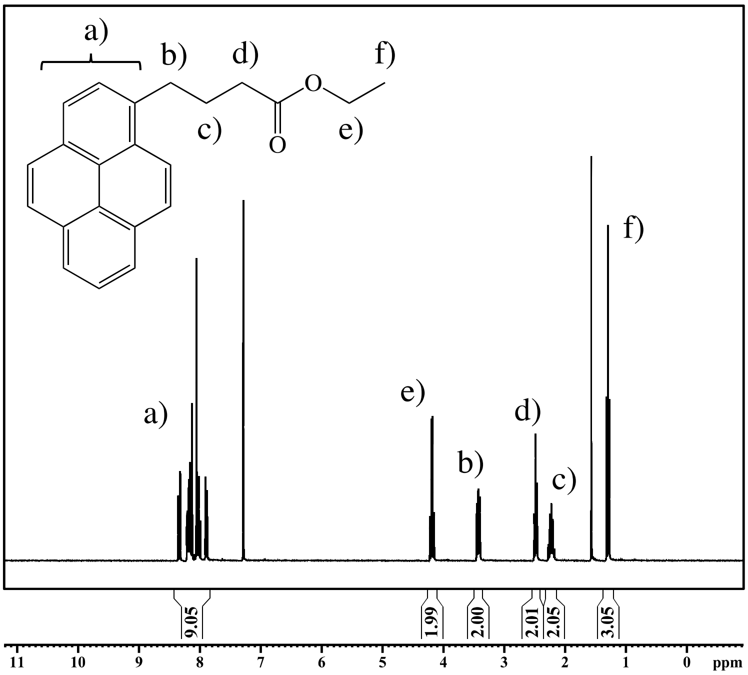

Figure S1. ^{1}H-NMR spectrum of ethyl 4-(1-pyrene)butyrate (PyBE). (300 MHz, $CDCl_3$): δ 1.30 (t, 3H), 2.23 (p, 2H), 2.48 (t, 2H), 3.42 (t, 2H), 4.18 (q, 2H), 7.82–8.41 (m, 9H).

Sample Fit of the MFA

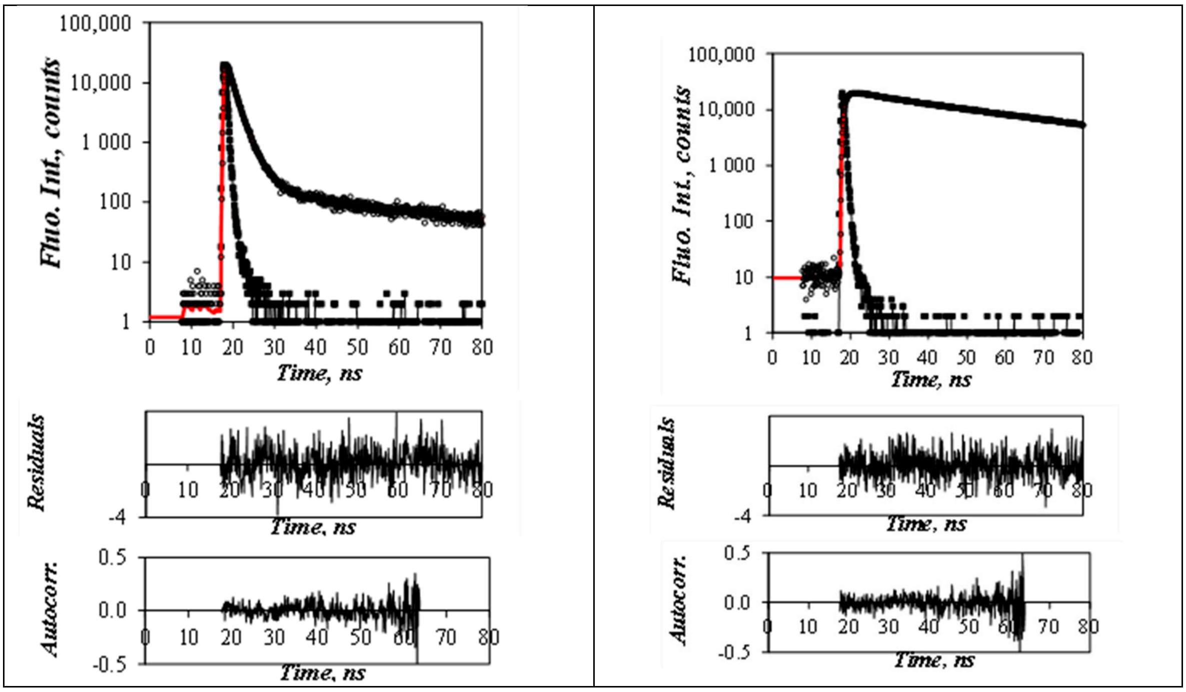

Figure S2. MFA of the (A) monomer (λ_{em} = 375 nm) and (B) excimer (λ_{em} = 510 nm) decay of Py_{32}-G(5) in degassed DMSO. χ^2 = 1.13, λ_{ex} = 344 nm.

SSF Spectra of Ethyl 4-(1-pyrene)butyrate (PyBE)

The SSF spectra for PyBE were acquired using the front face geometry to minimize reabsorption and the inner filter effect.

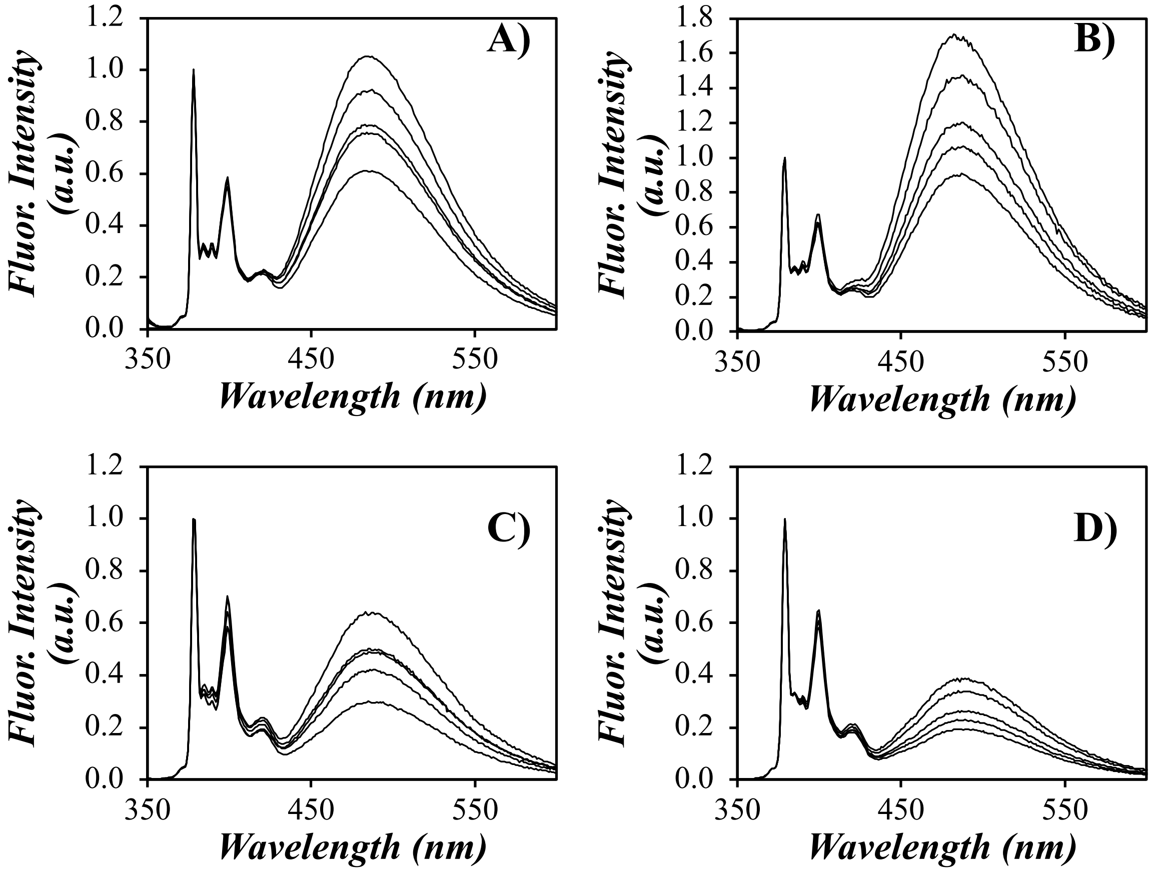

Figure S3. SSF spectrum of ethyl 4-pyrenylbutanoate in (A) THF, (B) toluene, (C) DMF, and (D) DMSO.

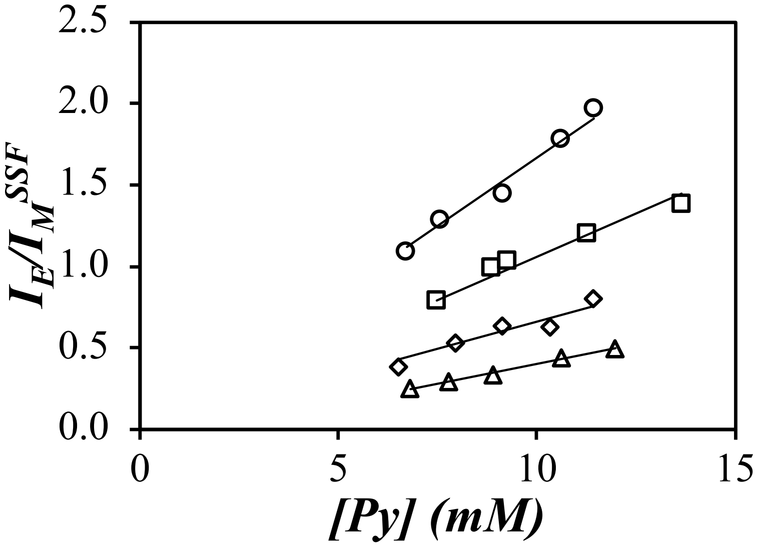

Figure S4. Plot of the I_E/I_M ratio versus concentration of PyBE in (○) toluene, (□) THF, (◇) DMF, and (△) DMSO.

Parameters Retrieved from the Global Birks Scheme Analysis

Table S1. Parameters retrieved from the global Birks scheme analysis of both the monomer and excimer decays of ethyl 4-(1-pyrene)butyrate in degassed toluene (Tol), *N,N*-dimethylformamide (DMF), dimethylsulfoxide (DMSO), and tetrahydrofuran (THF). Analysis program: globirks32bg.

	Conc. (mM)	a_{M1}	τ_1 *(ns)*	a_{M2}	τ_2 *(ns)*	a_{E1}	a_{E2}	k_{diff}×*[PyBE]* *(μs*$^{-1}$*)*	k_{-1} *(μs*$^{-1}$*)*	τ_E *(ns)*	χ^2
Tol τ_M = 194 ns	6.7	0.67	31	0.33	61	−2.27	2.29	22.0	2.7	53	1.07
	7.6	0.73	29	0.27	59	−1.88	1.90	24.8	2.6	52	1.19
	9.1	0.80	25	0.20	58	−1.65	1.66	30.3	2.7	52	1.16
	10.6	0.85	22	0.15	56	−1.54	1.56	36.0	2.7	52	1.22
	11.4	0.85	21	0.15	55	−1.49	1.50	38.1	3.0	51	1.21
DMF τ_M = 181 ns	6.5	0.28	42	0.72	81	−1.95	1.96	10.0	2.7	56	1.21
	8.0	0.40	40	0.60	73	−2.30	2.32	12.8	2.5	55	1.22
	9.1	0.45	38	0.55	68	−2.43	2.45	14.5	2.3	53	1.19
	10.3	0.54	36	0.46	65	−2.40	2.42	16.7	2.4	53	1.19
	11.4	0.59	34	0.41	63	−2.30	2.31	18.2	2.4	52	1.14
DMSO τ_M = 145 ns	6.8	0.18	43	0.82	82	−2.22	2.25	7.2	2.4	54	1.34
	7.8	0.20	43	0.80	75	−2.54	2.58	8.3	1.9	52	1.33
	8.9	0.26	42	0.74	70	−2.81	2.84	9.8	1.8	51	1.36
	10.6	0.36	42	0.64	64	−3.35	3.39	11.7	1.3	51	1.36
	12.0	0.39	41	0.61	62	−3.49	3.52	12.4	1.3	51	1.37
THF τ_M = 217 ns	7.5	0.50	31	0.50	76	−1.61	1.62	18.0	5.1	57	1.11
	8.9	0.57	29	0.43	72	−1.59	1.60	21.3	5.1	56	1.14
	9.3	0.60	29	0.40	70	−1.81	1.82	22.1	4.5	56	1.11
	11.3	0.68	26	0.32	66	−1.62	1.63	26.5	4.5	55	1.12
	13.7	0.75	23	0.25	63	−1.40	1.41	32.6	4.8	55	1.12

Parameters Retrieved from the MFA

Table S2. Parameters retrieved from the MFA (analysis program: sumegs10bg) of both the monomer and excimer decays of ethyl 4-(1-pyrene)butyrate in degassed tetrahydrofuran (THF), degassed toluene (Tol), degassed dimethylformamide (DMF), and degassed dimethylsulfoxide (DMSO).

	Conc. (mM)	a_1	τ_1 *(ns)*	a_2	τ_2 *(ns)*	f_{Ediff}^{E0}	f_{EE0}	τ_{E0} *(ns)*	χ^2
THF τ_M = 217 ns	7.5	0.46	29	0.54	75	1.00	0.00	57	1.03
	8.9	0.54	28	0.46	70	0.99	0.01	56	1.08
	9.3	0.58	28	0.42	68	0.99	0.01	56	1.05
	11.3	0.65	25	0.35	64	0.99	0.01	55	1.07
	13.7	0.72	22	0.28	61	0.99	0.01	55	1.02
Tol τ_M = 194 ns	6.7	0.64	30	0.36	60	0.99	0.01	53	1.02
	7.6	0.67	27	0.33	57	0.99	0.01	52	1.10
	9.1	0.74	24	0.26	53	0.99	0.01	52	1.11
	10.6	0.82	21	0.18	53	0.99	0.01	52	1.16
	11.4	0.81	20	0.19	50	0.99	0.01	51	1.14
DMF τ_M = 181 ns	6.5	0.21	37	0.79	79	1.00	0.00	56	1.10
	8.0	0.32	36	0.68	71	0.99	0.01	55	1.14
	9.1	0.31	32	0.69	64	0.99	0.01	53	1.07
	10.3	0.45	33	0.55	62	0.99	0.01	53	1.06
	11.4	0.45	31	0.55	58	0.99	0.01	52	1.05
DMSO τ_M = 145 ns	6.8	0.09	32	0.91	80	0.99	0.01	53	1.07
	7.8	0.10	31	0.90	73	0.99	0.01	51	1.18
	8.9	0.11	29	0.89	67	0.99	0.01	51	1.16
	10.6	0.13	29	0.87	60	0.99	0.01	51	1.01
	12.0	0.13	28	0.87	58	0.99	0.01	50	1.13

Table S3. Parameters retrieved from the MFA (analysis programs: sumegs14bg or sumegs33bg-4) of the monomer decays of the Py_x-G(*N*) dendrons in degassed toluene (Tol), degassed dimethylformamide (DMF), and degassed dimethylsulfoxide (DMSO).

	Generation (*N*)	a_1	τ_1 (ns)	a_2	τ_2 (ns)	a_3	τ_3 (ns)	*fMfree*	χ^2
Tol τ_M = 200 ns	1	0.16	1.6	0.79	5.7	0.04	17.2	0.010	1.05
	2	0.20	1.0	0.79	2.8	0.01	18.1	0.003	1.12
	3	0.25	0.4	0.73	1.7	0.01	7.5	0.004	1.02
	4	0.42	0.4	0.57	1.2	0.01	10.4	0.003	1.14
	5	0.57	0.3	0.42	1.0	0.01	6.9	0.003	1.17
	6	0.59	0.3	0.39	0.8	0.02	4.6	0.007	1.17
DMF τ_M = 180 ns	1	0.11	2.0	0.61	7.9	0.27	13.0	0.013	0.98
	2	0.24	2.0	0.74	4.3	0.01	32.9	0.004	1.01
	3	0.20	1.0	0.79	2.6	0.01	24.0	0.003	1.03
	4	0.50	1.2	0.50	2.1	0.01	30.1	0.002	1.04
	5	0.47	0.8	0.52	1.5	0.00	19.3	0.002	1.10
	6	0.87	0.8	0.12	1.8	0.01	15.9	0.003	1.09
DMSO τ_M = 145 ns	1	0.14	3.6	0.84	14.2	0.02	50.9	0.005	1.02
	2	0.19	2.0	0.79	6.3	0.01	26.8	0.003	1.01
	3	0.25	1.1	0.74	3.9	0.01	17.4	0.002	1.09
	4	0.40	1.3	0.59	3.1	0.01	22.0	0.002	1.07
	5	0.44	0.7	0.55	2.1	0.01	14.6	0.002	1.13
	6	0.61	0.6	0.38	1.6	0.01	14.8	0.002	1.13

Table S4. Parameters retrieved from the MFA of the excimer decays of the Py_x-G(*N*) dendrons in degassed toluene (Tol), degassed dimethylformamide (DMF), and degassed dimethylsulfoxide (DMSO).

	Generation (*N*)	f_{Ediff}^{E0}	f_{Ediff}^{D}	τ_{E0} (ns)	τ_D (ns)	τ_S (ns)	f_{EE0}	f_{ED}	f_{ES}^*	χ^2
Tol τ_M = 200 ns	1	0.95	-	49.4	-	4	0.00	-	0.05	1.05
	2	0.92	-	49.7	-	4	0.04	-	0.04	1.12
	3	0.88	-	49.0	-	4	0.04	-	0.08	1.02
	4	0.89	-	49.1	-	4	0.02	-	0.09	1.14
	5	0.89	-	49.6	-	4	0.01	-	0.09	1.17
	6	0.43	0.189	44.9	62.1	4	0.17	0.05	0.16	1.17
DMF τ_M = 180 ns	1	0.97	-	49.4	-	4	0.01	-	0.02	0.98
	2	0.95	-	49.4	-	4	0.01	-	0.05	1.01
	3	0.84	-	49.5	-	4	0.00	-	0.16	1.03
	4	0.79	-	49.4	-	4	0.00	-	0.21	1.04
	5	0.40	0.30	40.7	62.9	4	0.02	0.00	0.28	1.10
	6	0.44	0.08	42.5	116.0	4	0.16	0.00	0.32	1.09
DMSO τ_M = 145 ns	1	0.94	-	46.0	-	4	0.00	-	0.06	1.02
	2	0.93	-	45.9	-	4	0.02	-	0.05	1.01
	3	0.83	-	46.4	-	4	0.01	-	0.16	1.09
	4	0.77	-	46.4	-	4	0.00	-	0.23	1.07
	5	0.45	0.27	37.3	63.8	4	0.01	0.00	0.27	1.13
	6	0.35	0.25	33.2	60.3	4	0.00	0.13	0.28	1.13

Table S5. Molar fractions obtained from the MFA of the Py_x-G(*N*) dendrons in degassed toluene (Tol), degassed dimethylformamide (DMF), and degassed dimethylsulfoxide (DMSO).

	**Generation (*N*)**	f_{diff}^{E0}	f_{diff}^{D}	f_{diff}	f_{E0}	f_D	f_{agg}	f_{free}
Tol τ_M = 200 ns	1	0.99	-	0.99	0.00	-	0.00	0.01
	2	0.96	-	0.96	0.04	-	0.04	0.00
	3	0.95	-	0.95	0.05	-	0.05	0.00
	4	0.98	-	0.98	0.02	-	0.02	0.00
	5	0.98	-	0.98	0.01	-	0.01	0.00
	6	0.51	0.22	0.73	0.21	0.06	0.26	0.00
DMF τ_M = 180 ns	1	0.98	-	0.98	0.01	-	0.01	0.01
	2	0.99	-	0.99	0.01	-	0.01	0.00
	3	1.00	-	1.00	0.00	-	0.00	0.00
	4	1.00	-	1.00	0.00	-	0.00	0.00
	5	0.56	0.41	0.97	0.03	0.00	0.03	0.00
	6	0.64	0.12	0.76	0.23	0.01	0.24	0.00
DMSO τ_M = 145 ns	1	1.00	-	1.00	0.00	-	0.00	0.00
	2	0.98	-	0.98	0.02	-	0.02	0.00
	3	0.98	-	0.98	0.02	-	0.02	0.00
	4	1.00	-	1.00	0.00	-	0.00	0.00
	5	0.61	0.37	0.98	0.01	0.00	0.02	0.00
	6	0.48	0.34	0.82	0.00	0.17	0.17	0.00

© 2020 by the authors. Licensee MDPI, Basel, Switzerland. This article is an open access article distributed under the terms and conditions of the Creative Commons Attribution (CC BY) license (http://creativecommons.org/licenses/by/4.0/).
